# Comparison of aqueous humor cytokine and chemokine levels in diabetic patients with and without retinopathy

**Published:** 2012-04-04

**Authors:** Chui Ming Gemmy Cheung, Maya Vania, Marcus Ang, Soon Phaik Chee, Jing Li

**Affiliations:** 1 Ocular Inflammation and Immunology Services, Singapore National Eye Centre, Singapore; 2 Singapore Eye Research Institute, Singapore; 3 Department of Ophthalmology, Yong Loo Lin School of Medicine, National University of Singapore, Singapore; 4 Department of Ophthalmology, Xinhua Hospital, Shanghai Jiao Tong University School of Medicine, Shanghai, P.R. China

## Abstract

**Purpose:**

To compare the aqueous humor levels of proinflammatory and angiogenic factors of diabetic patients with and without retinopathy.

**Methods:**

Aqueous humor was collected at the start of cataract surgery from diabetic subjects and non-diabetic controls. The presence and severity of diabetic retinopathy were graded with fundus examination. Levels of 22 different inflammatory and angiogenic cytokines and chemokines were compared.

**Results:**

Aqueous humor samples from 47 diabetic patients (20 without retinopathy, 27 with retinopathy) and 24 non-diabetic controls were included. Interleukin (IL)-2, IL-10, IL-12, interferon-alpha (IFN-α), and tumor necrosis factor (TNF)-α were measurable in significantly fewer diabetic samples, and where measurable, were at lower levels than in non-diabetic controls. IL-6 was measurable in significantly more diabetic samples, and the median levels were significantly higher in the eyes with retinopathy than the eyes without retinopathy and the non-diabetic eyes. The vascular endothelial growth factor (VEGF) level was significantly higher in the diabetic eyes with and without retinopathy compared to the non-diabetic controls. The IL-6 level positively correlated with the monocyte chemotactic protein-1 (CCL2) and interleukin-8 (CXCL8) levels and negatively with the TNF-α level. The VEGF level negatively correlated with the IL-12 and TNF-α levels.

**Conclusions:**

The aqueous humor cytokine profile of diabetic patients without retinopathy was similar to that of patients with diabetic retinopathy. These cytokines may be useful biomarkers for early detection and prognosis of diabetic retinopathy. Compared to diabetic patients without retinopathy, only the IL-6 and VEGF levels were significantly higher in diabetic patients with retinopathy.

## Introduction

The role of angiogenic and proinflammatory cytokines in the development of diabetic retinopathy (DR) has been reported by many groups [1–13]. In particular, elevated levels of vascular endothelial growth factor (VEGF) have been observed in eyes with proliferative diabetic retinopathy (PDR) and diabetic macular edema (DME) [1–4]. Current treatment strategies aimed at inhibiting or reducing VEGF-related mechanisms are now either in use or being developed for treating DME and PDR. In addition to angiogenic factors, proinflammatory cytokines such as IL-1 and IL-6 have all been reported to be elevated in DME and PDR [3–8]. Elevated levels of chemokines, including interleukin-8 (CXCL8), interferon gamma-induced protein 10 (IP-10 or CXCL10), monocyte chemotactic protein-1 (MCP-1 or CCL2), and macrophage inflammatory protein-1β (MIP-1β or CCL4), have also been reported in PDR and have been suggested to play a role in attracting and activating leukocytes in inflammation [6,9,10]. Current studies on aqueous humor cytokines are limited due to the small volume of ocular samples and the inability to analyze a comprehensive range of cytokines and chemokines together. Recently, the multiplexed bead array assay has allowed simultaneous quantification of multiple factors in a small volume. However, to date, only a few studies investigating large combinations of cytokines or chemokines in DR have been published [13,14].

Most published studies on cytokine profiles have focused on eyes with PDR or DME [1–15]. Even in studies that included diabetic patients with mild or no DR, they were often used in the context as the control group for cases with more severe DR. However, there is histopathological evidence that retinal microangiopathy occurs in diabetic subjects well before the onset of retinal dysfunction and the appearance of clinically detectable retinopathy [16,17]. Functional tests have confirmed sub-clinical retinal and neuronal dysfunction in diabetic patients without retinopathy [18,19]. In this study, therefore, we compared the levels of cytokines in aqueous humor samples of eyes with DR compared to samples from eyes of diabetic patients without DR and healthy eyes of non-diabetic controls. The panel chosen is one of the most comprehensive among published studies in this area, allowing further study of correlations and interactions between the levels of cytokines and chemokines.

## Methods

This study was performed in accordance to the tenets of the Declaration of Helsinki and approved by the local Institutional Review Board (IRB) of the Singapore National Eye Centre. Consecutive patients listed for cataract surgery were invited to participate. Diagnosis of diabetes mellitus was self-reported. Patients with any other ocular condition (e.g., glaucoma, uveitis) apart from diabetic retinopathy were excluded. Informed consent was obtained from each study subject. The severity of retinopathy in diabetic subjects was graded with fundus examination at the time of listing and confirmed within one month after surgery with dilated fundoscopic examination. None of the patients developed macular edema post-op. Blood pressure and fasting blood glucose were measured before surgery. 47 diabetic and 24 non-diabetic patients were recruited, including 37 males and 34 females. Of the 47 diabetics, 27 had diabetic retinopathy and 20 had no retinopathy. The prevalence of coexisting hypertension and cardiovascular disease were also noted.

About 0.1–0.2 ml of aqueous humor was aspirated into a sterile tuberculin syringe at the beginning of cataract surgery after paracentesis was performed. Samples were quickly spun at 300× g for 5 min at 4 °C using a tabletop microcentrifuge and stored at –80 °C until further analysis.

### Cytokine analysis

Bio-Plex Pro^TM^ magnetic color-bead-based multiplex assay (Bio-Rad Laboratories, Inc., Hercules, CA) was used to measure the concentrations of 22 human cytokines/chemokines: Interleukin-1 receptor antagonist (IL-1Ra), IL-1β, IL-2, IL-4, IL-5, IL-6, IL-7, IL-10, IL-12, IL-13, IL-17, granulocyte colony-stimulating factor (G-CSF), granulocyte macrophage colony-stimulating factor (GM-CSF), interferon-gamma (IFN-γ), monocyte chemotactic protein-1 (CCL2), macrophage inflammatory protein-1β (MIP-1β or CCL4), interleukin-8 (CXCL8), monokine induced by interferon-gamma (MIG or CXCL9), interferon-gamma-induced protein 10 (IP-10 or CXCL10), tumor necrosis factor-alpha 2 (TNF-α), IFN-α, and vascular endothelial growth factor (VEGF). The assay was conducted according to the manufacturer’s instruction. Thirty-five microliters of aqueous humor sample was used in each reaction. Fluorescence intensity from the immunoassay was acquired and analyzed using the Bio-Plex^TM^ 200 System (software version 6.0; Bio-Rad Laboratories).

Concentrations lower than the low limit of detection were defined as non-measurable.

### Statistical analysis

Statistical analysis was performed using Predictive Analytics SoftWare (PASW) Statistics 18 (IBM Corporation, Armonk, NY). Statistical significance was accepted at p≤0.05. For categorical variables, the Pearson χ^2^ and Fisher’s Exact test analysis were performed. For numerical variables in parametric distribution, one-way ANOVA analysis was performed. For differences in cytokine concentrations, the Mann–Whitney U test with the Bonferroni correction was performed. The two-tailed, nonparametric Spearman method was used to assess the correlation between variables.

## Results

Aqueous humor samples were obtained from 47 diabetic patients (27 DR and 20 no-DR) and 24 non-diabetic controls. The baseline demographic data are summarized in Table 1. Grading of the severity of retinopathy at the time of surgery was as follows: mild non-proliferative (NPDR) in 15, moderate NPDR in two, severe NPDR in two, active proliferative (PDR) in one, and stable post-PRP (PRP was completed at least 12 months before surgery) in seven. Grading of retinopathy was further confirmed with dilated fundoscopy within the first month post-op. None of the patients had active clinically significant macular edema at the time of surgery. History of hypertension and cardiovascular disease were recorded. Blood pressure and fasting blood glucose measured at the start of surgery were collated. The mean age was not significantly different between the three groups: 64.9 years (22–89 years) among controls, 67.0 years (50–84 years) among the diabetic patients without retinopathy (No-DR) group, and 67.4 years (44–86 years) among the diabetic patients with retinopathy (DR) group.

**Table 1:** Baseline demographics of all patients.

	**Groups**			
**Characteristics**	**DR (n=27)**	**No-DR (n=20)**	**Control (n=24)**	**p-value**
Age (years), mean±SD	67.4±10.7	67.0±9.3	64.9±17.5	0.762^c^
**Gender, n (%)**				
Male	18 (66.7)	8 (40.0)	11 (45.8)	0.146^d^
Female	9 (33.3)	12 (60.0)	13 (54.2)	
Duration of diabetes (years), range^a^	4 - >30	1 - >10	N.A.	0.210^d^
Hypertension, n (%)	23 (85.2)	15 (75.0)	9 (37.5)	0.005^d^
Systolic BP	156.8±23.7	146±20.8	146.6±27.6	0.233^c^
Diastolic BP	73.8±13.0	74.3±11.3	78.5±13.2	0.400^c^
CVD, n (%)	9 (33.3)	6 (30.0)	2 (8.3)	0.110^d^
Blood glucose level (mM)^b^, mean±SD	9.0±2.8	8.2±2.9	5.1±1.1	0.018^c^

^a^4 groups: 0=No history of diabetes, 1=1–9 years, 2=10–19 years, 3=≥20 years ^b^fasting glucose level ^c^p-value by one-way ANOVA ^d^p-value by Pearson χ^2^ DR=Diabetic with retinopathy; No-DR=Diabetic with no retinopathy; BP=Blood pressure; CVD=Cardiovascular disease; SD=Standard deviation

The level of assayed cytokines and chemokines in each group is summarized in Table 2. IL-1β, IFN-γ, IL-4, IL-5, IL-17, and G-CSF were measurable in less than 50% of the samples from each group and therefore not included in the subsequent analysis.

**Table 2:** Summary of cytokine level in all measured aqueous humor.

	**DR n=27**		**No-DR n=20**		**Control n=24**		**p-value**		
**Cytokines**	**Median^a^**	**Range^b^**	**Median^a^**	**Range^b^**	**Median^a^**	**Range^b^**	**DR Control**	**No-DR Control**	**DR-No-DR**
IL-2	0.0*	0–3	0.0†	0–2	2.1	0–17	0.001	0.003	1.000
IL-13	2.2	0–75	0.6	0–11	2.8	0–8	1.000	1.000	0.981
IL-6	16.4*	0–1988	1.9	0–53	2.2	0–28	0.048	1.000	0.544
IL-10	0.0*	0–7	0.0†	0–1	1.3	0–3	0.003	0.004	1.000
IL-12	0.0*	0–2	0.0†	0–5	6.8	0–18	<0.001	0.001	1.000
TNF-α	0.0*	0–1	0.0†	0–4	7.6	0–15	0.002	0.019	1.000
IL-1Rα	76.5	0–185	9.3	8–414	8.6	3–96	0.414	0.634	1.000
IFN-α	0.0*	0–18	0.0†	0–20	26.2	0–84	0.008	0.012	1.000
CCL2	386.5*	21–1537	267.6	3–1863	193.1	76–816	0.030	0.645	1.000
CCL4	21.1	8–437	25.1	1–150	17.4	4–72	1.000	1.000	1.000
CXCL8	8.3	0–106	6.3	0–63	3	0–48	0.052	1.000	1.000
CXCL9	0	0–3243	0	0–493	89.5	0–1173	1.000	0.168	1.000
CXCL10	348.5	0–12055	104.7	0–1612	71.5	0–2571	1.000	1.000	1.000
IL-7	4.3	0–79	3.4	0–45	1.1	0–15	0.684	0.264	1.000
GM-CSF	0.0	0–309	0.0	0–416	125.4	0–562	0.071	0.824	1.000
VEGF	1587.3*	119–3375	781.0†	43–1287	39.8	25–81	<0.001	0.002	0.117

DR=Diabetic with retinopathy; No-DR=Diabetic with no retinopathy; IL=Interleukin; IFN=Interferon; TNF=Tumor necrosis factor; GM-CSF=Granulocyte-macrophage colony-stimulating factor; VEGF=Vascular endothelial growth factor. Significant difference is accepted at p<0.05. a Median of measured samples (pg/ml); b Range of measured samples (pg/ml); c p-value calculated using Mann–Whitney U with Bonferroni correction comparing two groups; * p<0.05, DR versus Control; † p<0.05, No-DR versus Control

**Figure 1:**
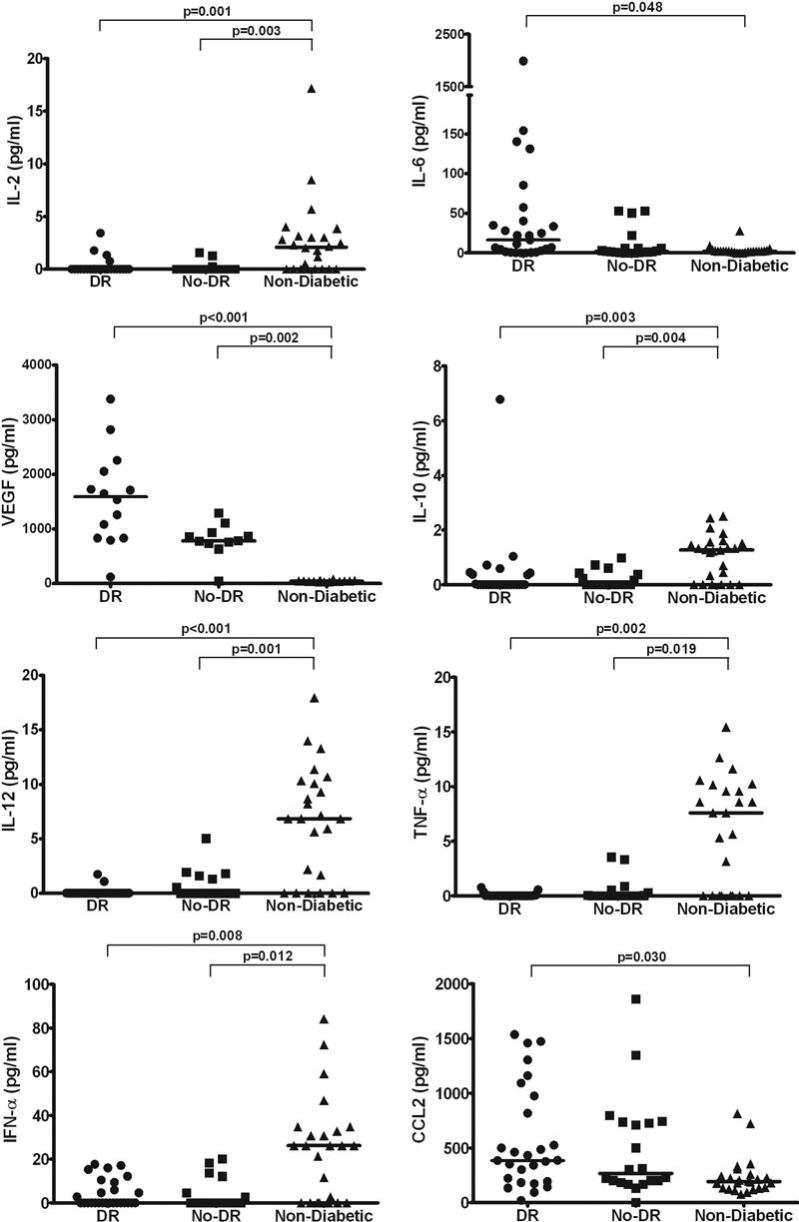
Scatter plots showing the distribution levels of IL-2, IL-6, VEGF, IL-10, IL-12, TNF-α, IFN-α, and CCL2 in aqueous humor from diabetic patients with retinopathy (DR, circles), diabetic patients without retinopathy (No-DR, squares), and non-diabetic controls (Non-Diabetic, triangles). The Mann–Whitney U test with the Bonferroni adjustment was conducted to compare two groups. Significant difference was accepted at p<0.05. Solid lines indicate the median.

Compared to non-diabetic controls, the percentage of samples from the diabetic patients reaching detectable concentrations of IL-2, IL-10, IL-12, IFN-α, TNF-α, and GM-CSF was significantly lower (Table 3). The median concentrations of these cytokines were also significantly lower in the DR and no-DR groups compared to the non-diabetic controls except for GM-CSF. IL-6 and IL-7 were detectable in a significantly higher percentage of the diabetic samples than in the non-diabetic controls. The median concentration of VEGF was significantly higher in the DR and no-DR groups compared to the non-diabetic controls. The median concentration of IL-6, CCL2, and CXCL8 was also significantly higher in the DR group (but not the no-DR group) compared to the non-diabetic controls (Table 2). For some cytokines, the concentration of individual sample in each group is shown in Figure 1.

**Table 3:** Total number of measurable aqueous samples analyzed by multiplexed-bead assay.

	**DR (n=27)**	**No-DR (n=20)**	**Control (n=24)**		**p-value^b^**
**Cytokines**	**%> LOD^a^**	**%> LOD^a^**	**%> LOD^a^**	**LOD^a^**	**DR and No-DR control**
IL-2	2/27	0/20	14/24	1.6	0.000
IL-13	17/27	8/20	17/24	0.7	0.153
IL-6	20/27	9/20	7/24	2.6	0.009
IL-10	8/27	5/20	18/24	0.3	0.000
IL-12	0/27	1/20	16/24	3.5	0.000
TNF-α	0/27	0/20	13/24	6	0.000
IL-1Rα	12/14	11/11	8/14	5.5	0.21
IFN-α	10/27	5/19	16/24	4.3	0.005
CCL2	27/27	20/20	24/24	1.1	-
CCL4	27/27	19/20	24/24	2.4	1.000
CXCL8	25/27	18/20	21/24	1	0.682
CXCL9	13/27	9/19	21/24	1.2	0.001
CXCL10	19/27	13/20	23/24	6.1	0.008
IL-7	19/27	15/20	10/24	1.1	0.012
GM-CSF	8/27	6/20	17/24	2.2	0.001
VEGF	14/14	11/11	14/14	3.1	0.680

DR=Diabetic with retinopathy; No-DR=Diabetic with no retinopathy; IL=Interleukin; IFN=Interferon; TNF=Tumor necrosis factor; GM-CSF=Granulocyte-macrophage colony-stimulating factor; VEGF=Vascular endothelial growth factor. Significant difference is accepted at p<0.05. ^a^Low limit of detection (pg/ml); ^b^p-value calculated using Pearson's Chi Square and Fisher's Exact test for %>LOD.

Ten subjects in the DR group had undergone previous panretinal photocoagulation (PRP). There was no significant difference in the level of any of the cytokines analyzed when compared with the remaining 17 subjects in the DR group who had never undergone PRP. Similarly, the presence of hypertension, cardiovascular disease, HbA1C level, and fasting blood glucose at the time of surgery was not associated with any significant alteration in any of the cytokine levels.

IL-6 was found to positively correlate with IL-7, CXCL8, IL-13, CCL2, and CCL4 in the DR and no-DR groups and negatively with TNF-α (Table 4). The VEGF level was negatively correlated with IL-12 and TNF-α. We did not find any correlation between IL-6 and VEGF.

**Table 4:** Correlation between IL-6 and VEGF with cytokines in DR and no-DR aqueous calculated using two-tailed Spearman correlation method. Significant difference is accepted at p<0.05.

**IL-6**	**TNF-α**	**GM-CSF**	**CCL2**	**CCL4**	**CXCL8**	**IL-7**	**IL-13**
	r_s_=-0.338	r_s_=-0.376	r_s_=0.744	r_s_=0.718	r_s_=0.855	r_s_=0.611	r_s_=0.725
	p=0.020	p=0.009	p=<0.001	p=<0.001	p=<0.001	p=<0.001	p=<0.001
VEGF	TNF-α	IL-12					
	r_s_=-0.448	r_s_=-0.469					
	p=0.025	p=0.018					

r_s_=Spearman coefficient.

## Discussion

In the current study, we compared the aqueous humor levels of cytokines and chemokines between diabetic patients with and without retinopathy and non-diabetic controls. We showed that the aqueous humor cytokine profile in diabetic patients without retinopathy closely mirrors that of patients with diabetic retinopathy and is significantly different compared to controls. IL-2, IL-10, IL-12, IFN-α, and TNF-α were unmeasurable in significantly more diabetic samples, and where measurable, the median levels were significantly lower in diabetic patients with and without retinopathy compared to non-diabetics. IL-6, on the other hand, was measurable in significantly more diabetic samples compared to non-diabetic controls. However, the median concentration was elevated only in those with retinopathy. VEGF levels were significantly higher in diabetic patients with and without retinopathy compared to non-diabetic controls. These alterations in cytokines are likely responsible for the pathogenesis of early stages of retinal damage due to diabetes. Understanding the role of all relevant cytokines in the pathogenesis of diabetic retinopathy is important for developing future therapeutic options.

Researchers have established that VEGF is one of the key cytokines responsible for the pathogenesis of PDR and diabetic macular edema [1–4]. Our results demonstrated progressively increasing VEGF levels from non-diabetic controls to the diabetic patients without retinography group, with the highest levels in the DR group. These results support that VEGF plays a significant role in the pathogenesis of DR well before the proliferative stage.

IL-6 is a multifunctional cytokine with proinflammatory properties. Elevated levels of IL-6 have been reported in PDR and DME [20–22]. In addition, IL-6 can induce VEGF expression. There have been contradictory reports regarding the correlation between IL-6 and VEGF levels in diabetic patients’ eyes [15,20–22]. In our study, IL-6 levels were significantly elevated only in eyes with retinopathy, and no correlation with VEGF levels was demonstrated. Our data suggest that in earlier stages of nonproliferative DR, the role of IL-6 may not be directly linked to VEGF. Indeed, IL-6 has been implicated in the breakdown of the blood-retinal barrier, which may contribute to the pathogenesis of earlier stages of nonproliferative diabetic retinopathy [23,24]. In addition, the correlation of IL-6 with multiple other cytokines suggest that it is a major driving force in causing the overall cytokine profile change in the aqueous humor of diabetic patients.

CCL2 and CXCL8 were elevated in eyes with diabetic retinopathy (but not in the diabetic patients without retinography group) compared to non-diabetic controls, in keeping with reports from previous studies [5–7,9,10,15,25]. In addition, we have demonstrated that IL-6 strongly correlates with chemokines in the diabetic group (r=0.74 for CCL2; r=0.86 for CXCL8), indicating that the increased levels are likely downstream effects of elevated IL-6 seen in eyes with retinopathy. CCL2 and CXCL8 are potent chemoattractants, in addition to being angiogenic, and are known to mediate leukocytic activation and adhesion to vascular endothelial cells. Such chronic leukocyte-mediated inflammation in the vascular walls may eventually lead to capillary occlusion and retinal ischemia.

IL-12 and TNF-α were negatively correlated with the VEGF level. The decreased level of IL-2, IL-10, IL-12, and TNF-α in the diabetic group compared to the non-diabetic controls is likely related to the increased VEGF levels in the former group. IL-10, IL-12, and TNF-α are major innate immunity mediators that also bridge the adaptive immune response. Decreased IL-12 is likely responsible for the decrease in IL-2 in the diabetic patients. We hypothesize that low concentrations of these cytokines in non-diabetic eyes provide an “immune vigilant” protection against potential assault. Disruption of such immune homeostasis is likely to be responsible for some of the pathological changes seen in diabetic eyes.

We also found reduced levels of IFN-α and GM-CSF in diabetic subjects compared to controls. IFN-α is a potent angiogenic inhibitor and has been shown to be effective in treating vascular tumors. Two pilot studies have suggested IFN-α may have a role in the regression of PDR and protect against retinal hemorrhage [26,27]. Our current results of reduced IFN-α levels in diabetic subjects further supports that this cytokine may have a protective role against retinal damage in diabetes. GM-CSF has been implicated in the retinal damage in uveitis [28]. This cytokine is an important regulator of macrophage, granulocyte, and dendritic cell behavior and function. TNF-α and IFN-γ have been shown to regulate GM-CSF production in retinal pigment epithelial cells. However, there is limited understanding of GM-CSF’s role in the pathogenesis of diabetic retinopathy.

Cytokine changes associated with diabetic retinopathy have been studied in serum [4], tears, vitreous [1–13], and aqueous humor [11,15,20,25]. More changes were observed in the vitreous as it closely reflects the pathological changes of the retina. In contrast to serum and tears, aqueous humor is likely to be a more accurate reflection of the intraocular milieu. The aqueous humor levels of VEGF and IL-6 have been compared with vitreous levels and were found to correlate strongly in diabetic retinopathy patients [20]. Furthermore, aqueous humor is readily harvested as part of routine cataract surgery, thus allowing the opportunity to study a larger number of subjects.

There are limitations to our study. These include the variable duration of diabetes and glycemic control and lack of information regarding other medical conditions. Some patients had received panretinal laser. However, there was no significant difference in any of the cytokine levels when patients in this group were separately compared with the remaining patients in the DR group. The levels of some cytokines showed large variations. We attempted to mitigate the large range by including in the analysis only cytokines that were measurable in at least 50% of controls. Furthermore, whether treatment for diabetes may alter aqueous humor cytokine levels is not known. In addition, as serum was not collected, we were not able to study the correlating serum levels of the cytokines reported. These cytokines might be increased in the serum of patients and enter the eye through leaky ocular blood barriers, and are not produced in the eye or have a function there. However, previous reports have found that ocular cytokine concentrations correlated poorly with serum levels [4,20,22]. Lastly, we cannot be certain that the aqueous cytokine profile from cataract controls would not differ from eyes without cataract.

In summary, we have demonstrated significant cytokine alteration in aqueous humor samples from diabetic patients without retinopathy. Despite the lack of clinically visible retinopathy, the cytokine profile of the aqueous humor from these eyes showed marked similarity to those with retinopathy, and was significantly distinct from the control eyes. These results lend further support that chronic inflammation plays a significant role in the pathogenesis of diabetic retinopathy, and that such inflammation likely occurs well before the development of any visible retinopathy. These cytokines may be future therapeutic targets for diabetic retinopathy in addition to VEGF.
